# Too much and too little: inborn errors of manganese homeostasis

**DOI:** 10.1186/s40348-026-00269-6

**Published:** 2026-11-13

**Authors:** Julien Park

**Affiliations:** Department of General Pediatrics, University Hospital Münster, Albert- Schweitzer-Campus 1, Geb. A1, Münster, 48149 Germany

**Keywords:** Manganese, Trace metal metabolism, Congenital disorders of glycosylation, Parkinsonism, Leigh syndrome, Inborn error of metabolism

## Abstract

Manganese is an indispensable cofactor for a wide range of enzymes in human metabolism, yet in excess it becomes toxic. Its homeostasis is therefore tightly controlled, and members of the solute carrier (SLC) family of transport proteins are central to this regulation. Over the past decade, inherited disorders of several manganese transporters have emerged as a distinct class of inborn errors of metabolism. Strikingly, pathogenic variants within this small group of transporters can disturb manganese balance in opposite directions: loss-of-function variants in *SLC30A10* and *SLC39A14* cause manganese overload, typically presenting with dystonia and parkinsonism, whereas variants in *SLC39A8* cause manganese deficiency and a congenital disorder of glycosylation. Importantly, and in contrast to most inborn errors of metabolism, several treatment approaches for these conditions already exist and are under active evaluation. This review summarizes the current understanding of the pathophysiology, clinical phenotypes, and therapeutic options for the inborn errors of manganese homeostasis.

## The physiology of manganese metabolism

Manganese is an essential trace element and an obligatory cofactor for numerous enzymes, including mitochondrial superoxide dismutase, arginase, glutamine synthetase, and the galactosyltransferases responsible for protein galactosylation. Because both deficiency and excess are harmful, and because manganese has no dedicated hormonal regulator of the kind that governs calcium or iron, its systemic levels are controlled almost entirely at the level of membrane transport, through the balance between intestinal absorption and hepatobiliary excretion. Dietary manganese is absorbed across the enterocyte [1], distributed in the circulation partly bound to transferrin and albumin [2], taken up by the liver [3], and ultimately eliminated into bile [4]. This reliance on a small repertoire of transporters, several of which are shared with iron, explains both the tight physiological set-point for manganese and the profound consequences of disrupting any single transporter.

**Fig. 1:**
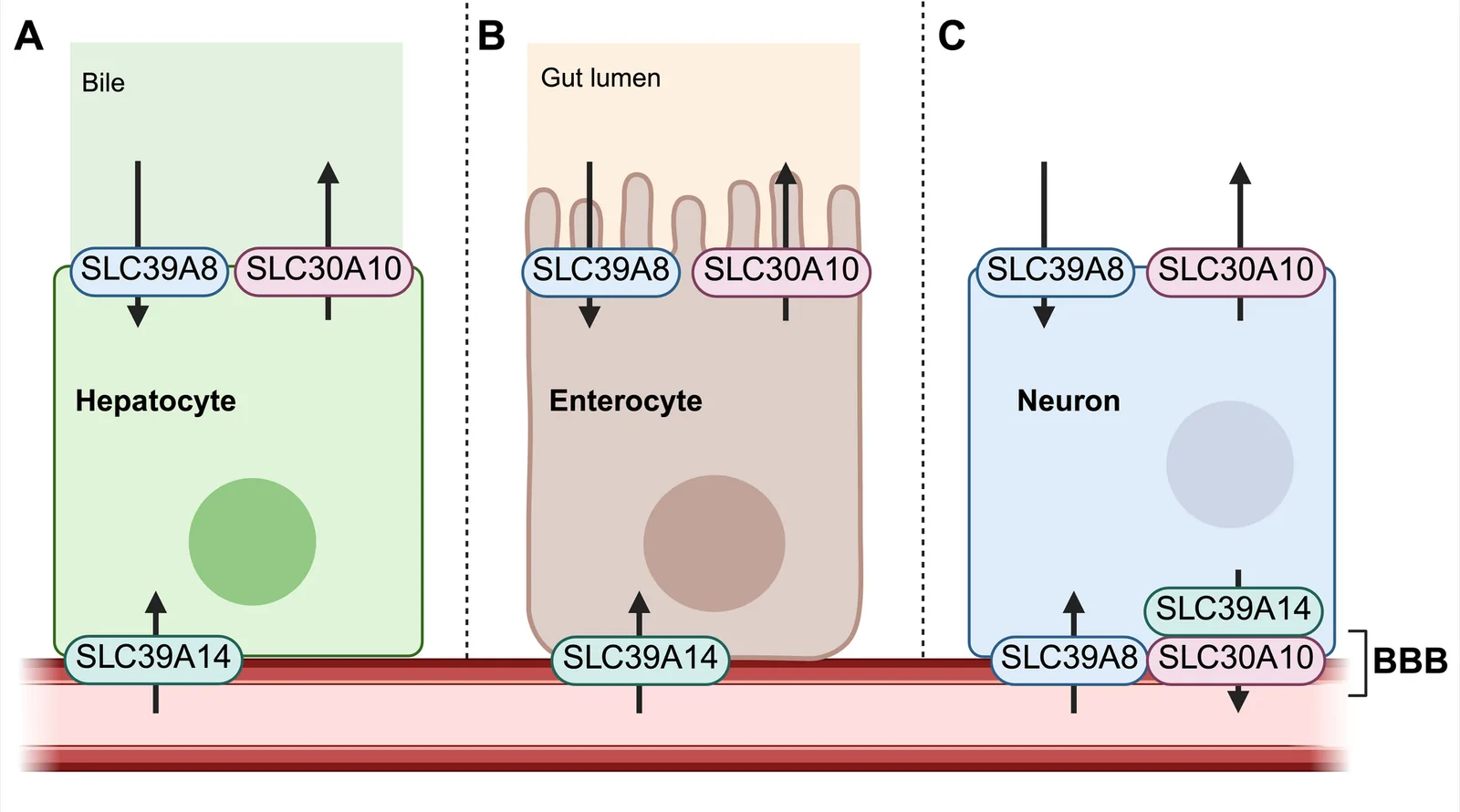
Overview of cellular manganese metabolism. Schematic of polarised manganese (Mn) transport in the three cell types that determine systemic Mn homeostasis. Arrows indicate the direction of net Mn flux mediated by each transporter. (**A**) Hepatocyte. SLC39A14 at the basolateral (sinusoidal) membrane clears Mn from the circulation into the hepatocyte, the obligatory first step of the biliary excretory route; SLC30A10 at the canalicular membrane then extrudes Mn into bile. Canalicular SLC39A8 reclaims Mn from bile, opposing SLC30A10-mediated export and retaining Mn for cellular use. (**B**) Enterocyte. SLC39A14 at the basolateral membrane mediates Mn uptake from blood, while SLC30A10 at the apical (brush-border) membrane extrudes Mn into the gut lumen for fecal loss; apical SLC39A8 mediates Mn uptake from the lumen, contributing to dietary absorption and to reclamation of Mn previously excreted by SLC30A10. (**C**) Brain. The lower membrane represents the blood–brain barrier (BBB), where SLC39A8 mediates Mn uptake from blood into the brain and SLC30A10 mediates efflux back into the circulation; SLC39A14 also contributes to Mn transport at this interface. The upper membrane represents the non-BBB neuronal surface, where SLC39A8 mediates Mn import into the neuron and SLC30A10 mediates Mn efflux. BBB, blood–brain barrier.Created in BioRender. Park, J. (2026) https://BioRender.com/5s13aq3

At the cellular level, manganese flux is directional and tissue-specific (Fig. 1). Import into enterocytes, hepatocytes, and other tissues is mediated principally by the divalent metal transporter DMT1 [5], the transferrin receptor [6], and two members of the ZIP (SLC39A) family, SLC39A8 (ZIP8) [1] and SLC39A14 (ZIP14) [7]. Export is the specialized function of the SLC30A (ZnT) family transporter SLC30A10 [8], which extrudes intracellular manganese across the canalicular membrane of hepatocytes and the apical surface of enterocytes into bile and the gut lumen.

The two ZIP importers both move manganese *into* cells, yet their deficiencies produce opposite phenotypes. The explanation lies in where each transporter sits and what the imported manganese is used for, not in the direction of transport itself. This is handily demonstrated by the role of each transporter in hepatic manganese metabolism, which determines systemic manganese homeostasis to a large extent:

SLC39A14 is expressed on the basolateral (sinusoidal) membrane of the hepatocyte, where it clears manganese from portal and systemic blood into the liver. Hepatic uptake is the obligatory first step of the biliary excretory route, because manganese must enter the hepatocyte before SLC30A10 can pass it into bile. Loss of SLC39A14 therefore blocks excretion at its entry point: manganese cannot leave the circulation, accumulates in blood, and is deposited in the brain, producing hypermanganesemia with dystonia (HMNDYT2) [9, 10].

SLC39A8 operates in the service of cellular acquisition and conservation rather than excretion. Its loss impairs delivery of manganese to the cells and enzymes that require it, and the resulting phenotype is systemic manganese deficiency (hypomanganesemia), with impaired activity of manganese-dependent enzymes such as β-1,4-galactosyltransferase and manganese superoxide dismutase [11].

The organizing principle is thus the position of each transporter within the excretory pathway. Defects that interrupt the route out of the body cause overload, whether at the final export step (SLC30A10) or at the hepatic uptake step that precedes it (SLC39A14), whereas a defect in import for cellular use (SLC39A8) causes depletion. This architecture explains why three disorders united by a common metal diverge so markedly in their biochemical and clinical signatures (Table 1).

**Table 1:** Clinical comparison of disorders of manganese homeostasis

Feature	SLC30A10 deficiency	SLC39A14 deficiency	SLC39A8 deficiency
**OMIM**	#613​280	#617​013	#616​721
Gene / Protein	SLC30A10 / ZnT-10 (Mn exporter)	SLC39A14 / ZIP14 (Mn importer)	SLC39A8 / ZIP8 (Mn importer)
Inheritance	Autosomal recessive	Autosomal recessive	Autosomal recessive
Mn direction	Manganese excess	Manganese excess	Manganese deficiency
Blood Mn	Markedly elevated	Markedly elevated	Low to undetectable
Age of onset	1–57 years (mostly childhood; rare adult-onset)	0.5–18 years (childhood)	Infancy, childhood
Motor features	Dystonia, “cock-walk” gait, truncal ataxia, bradykinesia, parkinsonism	Dystonia, spasticity, limb contractures, resting tremor, bradykinesia, loss of ambulation	Hypotonia, dystonia, limb stiffness, spasms
Cognitive	Mild cognitive impairment	Mild cognitive impairment	Moderate-to-profound intellectual disability
Seizures	Uncommon	Uncommon	Prominent (infantile spasms/epileptic encephalopathy)
Liver disease	Present — steatosis → fibrosis → cirrhosis	Absent	Elevated transaminases (variable)
Polycythemia	Present	Absent	Absent
Iron stores	Depleted (iron deficiency)	Normal	Normal
Glycosylation	Normal	Normal	Abnormal— type II CDG pattern (hypogalactosylation)
Other systemic features	Hypothyroidism (reported)	None characteristic	Cranial asymmetry/craniosynostosis, short stature, sensorineural hearing loss, strabismus, cortical blindness
Brain MRI	T1 hyperintensity of basal ganglia and white matter; T2 hypointensity of basal ganglia	T1 hyperintensity of basal ganglia, white matter, and cerebellum; T2 hypointensity of basal ganglia	T2 hyperintensity of basal ganglia (Leigh-like); cerebellar atrophy; T1 hypointensity
Levodopa response	Poor	Poor	Not applicable
Primary treatment	CaNa₂-EDTA chelation + iron supplementation	CaNa₂-EDTA chelation	Oral manganese supplementation
Treatment response	Variable; can stabilize/improve neurological and hepatic disease if started early	Variable; can lower Mn and produce clinical improvement	Good — complete normalization of glycosylation; clinical improvement in motor, hearing
Prognosis without treatment	Potentially fatal (progressive cirrhosis)	Progressive neurodegeneration, loss of ambulation	Severe disability; potentially fatal

## Disorders of manganese excess

### SLC30A10 deficiency

The first recognized manganese accumulation disorder, SLC30A10 deficiency, also called hypermanganesemia with dystonia type 1, HMNDYT1, or the syndrome of hepatic cirrhosis–dystonia–polycythemia–hypermanganesemia, “CDPH”, MIM #613280) was initially described clinically [12] before the underlying genetic defect was identified simultaneously by two independent groups [13, 14]. It is caused by biallelic loss-of-function variants in *SLC30A10*, which encodes a manganese efflux transporter expressed at the *canalicular* membrane of hepatocytes, in enterocytes, and in neurons, where it mediates the export of intracellular manganese into bile and the intestinal lumen as well as its excretion across the blood-brain-barrier (BBB) [15]. Disruption of this transporter abolishes the principal route of manganese excretion, producing systemic manganese overload with preferential deposition in the basal ganglia. The disorder is defined by a characteristic triad of systemic features - chronic liver disease, polycythemia, and depletion of iron stores - that accompanies the neurological phenotype and provides important diagnostic clues [12–14].

The clinical presentation is notably heterogeneous in its timing. A childhood-onset form typically emerges within the first decade, manifesting with progressive dystonia, gait disturbance, and a high-stepping, toe-walking “cock-walk” gait, frequently combined with dysarthria, fine tremor, and spastic paraparesis. An adult-onset form, by contrast, is dominated by parkinsonian features, i.e. bradykinesia, rigidity, and hypomimia [16]. In both forms the movement disorder is largely pharmacoresistant, and sustained abnormal posturing may culminate in secondary musculoskeletal complications and progressive loss of independent function [12, 16]. Hepatic involvement is present from early in the course and evolves from hepatomegaly and transaminase elevation toward fibrosis and, ultimately, cirrhosis with its attendant complications, constituting a principal source of morbidity and, in untreated disease, of premature mortality [17].

Biochemical evaluation reveals markedly elevated whole-blood manganese, frequently exceeding several times the upper limit of normal and serving as the diagnostic hallmark. This is accompanied by polycythemia, elevated liver transaminases, and a distinctive iron profile of low serum iron and ferritin with raised total iron-binding capacity, reflecting depleted iron stores. Critically, serum copper and ceruloplasmin remain normal, distinguishing the condition from Wilson disease [18]. On neuroimaging, brain MRI characteristically demonstrates symmetrical T1-weighted hyperintensity within the basal ganglia, most conspicuously the globus pallidus and striatum, attributable to the paramagnetic properties of deposited manganese, with corresponding T2-weighted signal that is typically normal or hypointense; signal change may extend to the white matter, dentate nuclei, and anterior pituitary [13, 17].

Therapy is directed at reducing the cerebral and systemic manganese burden, since the underlying transporter defect cannot currently be corrected. The cornerstone is intravenous chelation with disodium calcium edetate (CaNa₂EDTA), delivered in intermittent cycles to mobilize manganese and substantially augment its urinary elimination, thereby lowering whole-blood manganese concentrations, ameliorating MRI signal abnormalities, and improving both neurological and hepatic indices. Iron depletion is treated with oral iron supplementation. This is additionally beneficial since iron and manganese compete for shared transport pathways [19]. Clinical benefit hinges on timing, with early initiation offering the greatest prospect of stabilizing or improving the phenotype, whereas late intervention may normalize manganese biochemically with limited effect on existing deficits. In addition, supportive and symptomatic care with dystonia management, mototherapy, and nutritional support is imperative.

Consensus statements for the management of SLC30A10 and SLC39A14 deficiency (see below) have recently been published [20].

### SLC39A14 deficiency

First described by Tuschl and colleagues in 2016 [21], SLC39A14 deficiency (also called hypermanganesemia with dystonia type 2, HMNDYT2; MIM # 617013) presents as a childhood-onset, progressive parkinsonism-dystonia. Caused by biallelic loss-of-function variants in *SLC39A14*, the clinical picture is marked by an initial phase with loss of developmental milestones, gait disturbance, and prominent axial hypotonia typically at the age of six months to three years [22, 23]. Importantly, liver function is preserved and no hematological abnormalities are present [21]. This difference to SLC30A10 deficiency can be explained by where each transporter acts. SLC30A10 exports manganese out of the hepatocyte into bile, so its loss traps the metal inside the liver and injures it. SLC39A14 works one step earlier, carrying circulating manganese into the hepatocyte in the first place; when it is lost, manganese never enters the liver, sparing the organ but remaining in the blood, from where it is redistributed to the brain. The block therefore shifts the manganese burden away from the liver and onto the nervous system, accounting for hypermanganesemia and neurotoxicity without hepatic or hematological disease.

In the later phase of the disorder, typically emerging toward the end of the first decade of life, the clinical picture is dominated by severe, generalized, and largely pharmacoresistant dystonia that supersedes the earlier hypotonic phase, frequently combined with worsening spasticity and parkinsonian features such as bradykinesia, rigidity, and hypomimia. Sustained abnormal posturing leads to progressive secondary musculoskeletal complications, including fixed limb contractures and scoliosis, and most affected individuals lose independent ambulation and become dependent for daily activities. Bulbar dysfunction, present from earlier in the disease, deteriorates markedly, with advancing dysarthria progressing toward severely impaired or unintelligible speech and worsening dysphagia that predisposes to aspiration, recurrent respiratory infections, and compromised nutrition, sometimes necessitating gastrostomy feeding; bulbar and respiratory compromise are key contributors to morbidity and, in untreated disease, to premature mortality. A clinically striking feature throughout this stage is the relative preservation of cognition despite profound motor and bulbar disability, leaving affected individuals aware while severely physically impaired [21, 24].

In SLC39A14 deficiency, the defining biochemical abnormality is a marked elevation of whole-blood manganese, typically several-fold above the reference range, which serves as the key diagnostic marker. As stated previously, liver function remains intact [21].

Brain MRI characteristically demonstrates symmetrical T1-weighted hyperintensity and corresponding T2-weighted hypointensity within the basal ganglia, particularly the globus pallidus and striatum, owing to the paramagnetic properties of deposited manganese. Signal change frequently extends to involve the dentate nuclei, the anterior pituitary, the white matter, and the brainstem, while the posterior limb of the internal capsule may also be affected [21].

Therapy for SLC39A14 deficiency centers on reducing the brain and systemic manganese burden, since there is no means of correcting the underlying transporter defect. The mainstay is intravenous chelation with CaNa_2_EDTA, administered in intermittent cycles, which mobilizes manganese and markedly increases its urinary excretion, thereby lowering whole-blood manganese levels. Because chelation can also deplete iron [25] and because iron and manganese compete for shared transport pathways [19], treatment is often combined with oral iron supplementation to further limit manganese uptake and prevent iatrogenic iron deficiency. The clinical benefit is highly dependent on timing: early initiation, before the fixed, generalized dystonia and musculoskeletal complications of the later phase become established, offers the best prospect of stabilizing or improving the neurological picture, whereas treatment begun late may lower manganese levels biochemically with little reversal of entrenched deficits [21, 24]. Alongside this disease-modifying approach, supportive and symptomatic care, including management of dystonia and spasticity, physiotherapy, nutritional support with safe feeding (including gastrostomy where dysphagia threatens aspiration), and respiratory care, remains essential.

## Disorders of manganese deficiency

### SLC39A8 deficiency

Biallelic pathogenic variants in *SLC39A8* were recognized as the cause of a severe neurodevelopmental phenotype in parallel by two independent groups in 2015 [26, 27]. The resulting disorder—formally designated SLC39A8-CDG (congenital disorder of glycosylation type IIn; MIM #616721)—arises from impaired ZIP8 function, with these variants producing intracellular manganese depletion and systemic manganese deficiency in affected individuals [27]. Because manganese serves as a cofactor for multiple enzymes, its depletion results in multisystem consequences in which neurological manifestations constitute the defining phenotypical feature.

SLC39A8 deficiency typically presents in infancy as a severe, multisystem disorder in which neurological features dominate. The core manifestations are developmental delay and intellectual disability (ranging from mild to profound), axial hypotonia, dystonia, spasticity, and epilepsy, often accompanied by feeding difficulties, poor weight gain, and growth deficiency [27, 28]. A characteristic that sets the disorder apart from the hypermanganesaemic conditions is involvement of the special senses, with sensorineural hearing impairment and ophthalmologic abnormalities including strabismus and cortical blindness. Additional features reported in the index cohorts include cranial asymmetry, infantile spasms with hypsarrhythmia, and disproportionate short stature [27]. Importantly, however, the clinical spectrum is not uniformly severe: milder presentations also occur, in some instances amounting to isolated ataxia, and awareness of this attenuated end of the spectrum is essential if such cases are not to be overlooked.

In contrast to the hypermanganesemic disorders SLC30A10 and SLC39A14 deficiency, the biochemical hallmark of SLC39A8 deficiency is a strikingly low, at times even undetectable, whole-blood manganese, reflecting impaired cellular uptake of the metal [27]. Because several enzymes central to protein glycosylation - most notably the galactosyltransferases - depend on manganese as an essential cofactor, this manganese deficiency translates into defective glycosylation, and the condition is accordingly classified among the congenital disorders of glycosylation (CDG) [27, 29, 30]. Abnormal glycosylation is therefore a characteristic feature, yet it may be overlooked by conventional first-line screening, since analysis of serum transferrin by high-performance liquid chromatography (HPLC) or comparable methods can appear normal; more refined techniques, such as MALDI-TOF mass spectrometry, are frequently required to disclose the underlying glycosylation defect [31].

In addition to glycosylation abnormalities, patients may exhibit substantial impairment of mitochondrial function with clinical features reminiscent of Leigh syndrome, i.e. severe developmental delay, dystonia, and seizures. Cranial MRI typically shows basal ganglia T2-hyperintensities in addition to cerebral and cerebellar atrophy [32]. Biochemically, lactate elevation and impairment of several respiratory chain complexes can be observed. The mitochondrial manifestations of the disorder are attributed to impaired function of the manganese dependent superoxide dismutase 2 (SOD2, MnSOD) [33]. Given that glycosylation abnormalities represent only one facet of the pathomechanism the classification of SLC39A8 deficiency as a CDG can be questioned. The dysglycosylation is a downstream consequence of disturbed manganese homeostasis rather than a defect in a glycosylation pathway per se, a distinction reflected in the observation that SLC39A8 deficiency is primarily a disorder of manganese metabolism that results in secondary hypoglycosylation. Accordingly, it may be more accurate to regard SLC39A8 deficiency as a disorder of manganese transport with secondary glycosylation abnormalities, a view further supported by the fact that manganese supplementation, rather than any glycosylation-directed intervention, addresses the root cause of the disease.Although manganese serves as a cofactor to additional enzymes beyond SOD2 and galactosyltransferases, there is currently insufficient evidence to indicate clinically relevant impairment of the affected pathways, including the urea cycle, purine metabolism, and gluconeogenesis.

Manganese substitution is the mainstay of treatment in SLC39A8 deficiency. Treatment with Mn-II-sulfate was shown to achieve biochemical correct with normalized manganese levels as well as glycosylation in treated patients [34]. To date, no systematic evaluations of varying manganese doses have been performed. Based on the experience of a cohort of thirty patients, titration of manganese supplementation with a starting dose of 0.8 mg elemental manganese / kg bodyweight /day has been proposed. Untreated, the disorder follows a severe course commensurate with its early onset and multisystem burden; the recognition that targeted manganese repletion can achieve both biochemical correction and major clinical improvement [34], however, has transformed the outlook, reinforcing that timely diagnosis and initiation of therapy are decisive determinants of outcome.

## Does the genotype predict the phenotype in disorders of manganese homeostasis?

Current evidence indicates that both hypomorphic (missense) and null (truncating or frameshift) variants are unequivocally disease-causing in SLC39A8, SLC30A10, and SLC39A14 deficiencies, yet there is no clear correlation between variant class and either age of onset or clinical severity.

For example, the SLC30A10 p.Thr196Profs17 (c.585del) frameshift variant has been reported in individuals with markedly different presentations: one patient developed isolated erythrocytosis and mild dystonia at age 7, while another with the same homozygous variant presented at age 14 with spastic paraparesis, hepatomegaly, and cirrhosis, illustrating substantial variability even with identical genotypes [13]. Similarly, the SLC30A10 p.Gln412Argfs26 (c.1235delA) frameshift variant was identified in two Italian brothers who both presented unusually late, at ages 47 and 57, with progressive parkinsonism and polycythemia, a much later onset than typically seen in this disorder [14]. In SLC39A14 deficiency, siblings homozygous for the p.Leu353Arg (c.1058T > G) missense variant showed differences in age of onset, cognitive impairment, and degree of motor regression, with one sibling presenting at 18 months with rapid deterioration and severe cognitive impairment, and the other at 3 years with milder and more slowly progressive symptoms [21]. Likewise, in SLC39A8 deficiency, there is considerable difference in the severity of disease observed in indviduals homozygous for the G38R variant [26]. These examples underscore that no clinically relevant genotype-phenotype correlations have been established for SLC39A8, SLC30A10, or SLC39A14 deficiencies. While multiple mechanisms such as the influence of modifier genes, dietary and environmental manganese exposure, iron status (which can affect manganese absorption), hepatic function, and ascertainment bias due to small sample sizes might explain these findings, the lack of correlation between genotype and phenotype remains imperfectly understood. Further research on these modifying factors might facilitate innovative treatment approaches for these devastating disorders.

## Diagnostic approach

**Fig. 2:**
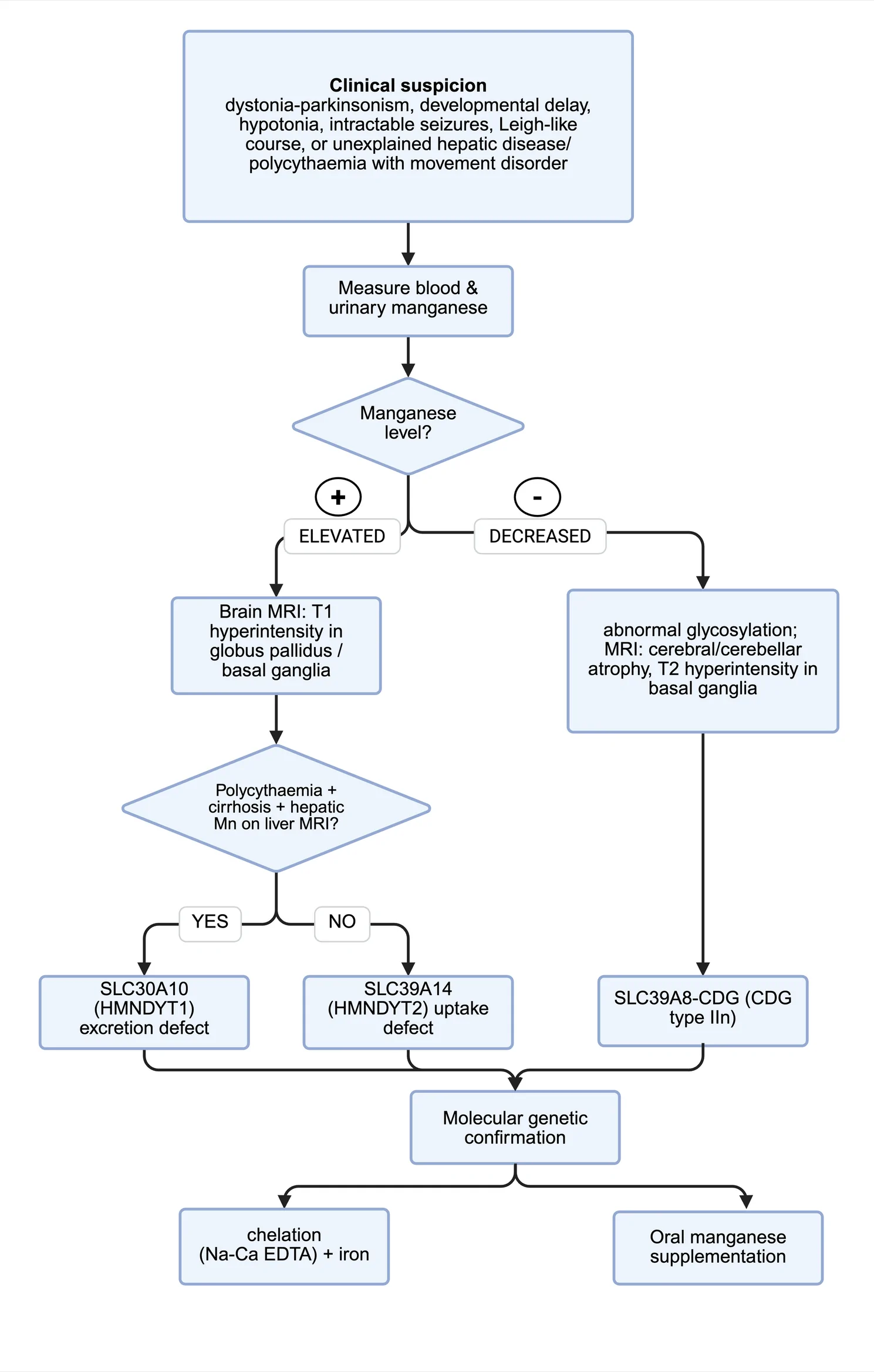
Diagnostic algorithm for inborn errors of manganese metabolism. Starting from clinical suspicion, the algorithm uses blood and urinary manganese levels, MRI findings, and additional clinical features to distinguish the three disorders and guide molecular confirmation and treatment. CDG, congenital disorder of glycosylation; EDTA, ethylenediaminetetraacetic acid; HMNDYT, hypermanganesaemia with dystonia; Mn, manganese; MRI, magnetic resonance imaging. Created in BioRender. Park, J. (2026) https://BioRender.com/s0iwnfk

Because the three inherited disorders of Mn metabolism diverge into biochemically opposite categories of Mn overload and Mn deficiency, circulating and urinary Mn concentrations provide the pivotal early discriminator around which diagnostic evaluation should be organized. The following sequential, biomarker-anchored algorithm may guide clinicians from initial clinical suspicion to genotype-directed management (Fig. 2).

### Step 1 — clinical recognition

A disorder of Mn metabolism should be considered in children presenting with early-onset dystonia or dystonia-parkinsonism and a characteristic gait disturbance, or with unexplained developmental delay, hypotonia, growth deficiency, and seizures—including infantile spasms with hypsarrhythmia. The combination of a movement disorder with polycythemia and/or chronic liver disease is a particularly strong pointer to the overload disorders, whereas a Leigh-like neurodevelopmental course favors SLC39A8 deficiency.

Before pursuing a monogenic diagnosis, acquired causes of manganese accumulation must be excluded, since they are considerably more common than the inherited disorders and produce an identical biochemical and radiological signature. Because manganese is eliminated almost exclusively via the hepatobiliary route [4], any condition that impairs biliary excretion or bypasses hepatic clearance can raise whole-blood manganese and generate symmetrical T1-weighted pallidal hyperintensity indistinguishable from that of the overload disorders. The principal acquired causes are chronic cholestatic or end-stage liver disease, portosystemic shunting (congenital or acquired), and prolonged parenteral nutrition, in which manganese is delivered directly into the circulation while enteral regulation is bypassed; environmental or occupational overexposure (e.g. welding fumes, contaminated water) should also be considered in older children and adolescents. A careful history, assessment of hepatic and vascular anatomy, and review of nutritional support will usually distinguish these secondary states from a primary transporter defect, and their identification is important because manganese biochemistry and MRI changes are frequently reversible once the underlying source is corrected.

### Step 2 — first-line biochemical screening

Quantification of whole-blood and urinary manganese is the decisive initial investigation and directs all subsequent triage. This should be accompanied by a full blood count (to detect polycythemia), liver function tests, and iron studies. Where deficiency is suspected, glycosylation analysis should be added - ideally mass-spectrometry-based glycome analysis, the current diagnostic standard for SLC39A8-CDG, which characteristically reveals defective terminal galactosylation and sialylation.

### Step 3 — neuroimaging and organ assessment

Brain MRI demonstrating T1-weighted hyperintensity within the globus pallidus and basal ganglia is the imaging hallmark of Mn overload. Liver MRI is a key discriminator between the two overload disorders, as hepatic Mn accumulation and cirrhosis characterize SLC30A10 disease but are absent in SLC39A14 disease. In SLC39A8 deficiency, imaging instead typically reveals cerebral and cerebellar atrophy without Mn-related signal change. Individuals exhibiting the mitochondrial phenotype of SLC39A8 deficiency may exhibit T2-weighted hyperintensities of the basal ganglia.

### Step 4 — molecular confirmation

Definitive diagnosis is established by genetic analysis of *SLC30A10*, *SLC39A14*, and *SLC39A8*. Comprehensive, next-generation based sequencing analyses are strongly encouraged to avoid diagnostic delays.

This structured work-up will allow the identification of the currently known disorders of manganese metabolism by means of clinical, biochemical, and imaging findings (Fig. 2). It should be emphasized that the proposed algorithm represents a logical rather than a strictly chronological framework. In practice, most patients will already have undergone neuroimaging, and frequently exome or genome sequencing, before a disorder of manganese homeostasis is considered. Manganese quantification, by contrast, is not routinely performed and is restricted to specialist laboratories; it is therefore frequently obtained late, as a confirmatory and baseline measurement. We nevertheless position it early because it is the most efficient discriminator between overload and deficiency states and because it permits treatment to be initiated before molecular confirmation is available. Entry into the algorithm may occur at any step, and the sequence should be adapted to the presenting phenotype and to local diagnostic resources.

## Future directions and conclusions

The inherited disorders of manganese transport constitute a compelling illustration of how disruptions in the metabolism of a single trace element can produce divergent, and at times diametrically opposed, biochemical and clinical phenotypes. Beyond their intrinsic clinical importance, these monogenic conditions have been instrumental in shaping our understanding of manganese metabolism in both health and disease: it was through the identification and characterization of these transporter defects that the physiological architecture of manganese metabolism was clarified in humans. In this respect the disorders serve as natural experiments, each loss-of-function state illuminating the specific homeostatic role of the transporter concerned. Equally significant is the recognition that they are among the few genetic neurodegenerative diseases that are directly treatable, and that clinical outcome is strongly contingent on the timeliness of intervention.

This therapeutic time-dependence makes timely diagnosis highly relevant. A crucial and readily actionable message is that the analysis of manganese is straightforward, inexpensive, and rapidly performed: whole-blood manganese can be measured with widely available methods and is grossly abnormal in these conditions. Given the low burden of the test against the high cost of a missed, treatable diagnosis, whole-blood manganese estimation should be adopted as a low-threshold investigation in any patient presenting with atypical dystonia or ataxia, particularly where the clinical picture is progressive, early in onset, or otherwise unexplained. Such a low-threshold approach offers the most practical route to identifying these rare but modifiable conditions before fixed neurological and musculoskeletal deficits become established.

Notwithstanding these advances, our understanding of manganese metabolism remains conspicuously incomplete. A striking feature of the field is that several transporters appear to mediate ostensibly overlapping functions at the same anatomical site – multiple importers, for instance, operating in parallel within the same cell – yet the distinct, non-redundant contribution of each individual transporter is still poorly defined. Why the system should be so apparently redundant, and under what circumstances one transporter becomes rate-limiting or irreplaceable, are questions that current models do not fully answer. Compounding this uncertainty is the considerable phenotypic variability observed among affected individuals, including differences in age of onset, disease progression, and organ involvement that are not readily explained by genotype alone. Such heterogeneity strongly implies the existence of modifying factors that have yet to be identified, and their elucidation represents a priority for future mechanistic and cohort-based study.

Therapeutic development, likewise, holds both promise and unresolved challenges. Current management of the overload disorders rests on intravenous chelation with disodium calcium edetate combined with oral iron supplementation, an approach that is effective biochemically but burdensome to deliver and often incomplete in reversing entrenched deficits, underscoring the need for optimised, and ideally less onerous, chelation strategies. For SLC39A8 deficiency, in which the fundamental defect is one of manganese deficiency rather than excess, treatment centers on manganese repletion, and here a particular caution deserves emphasis. Galactose substitution should not be regarded as a benign adjunct: because galactose can restore glycosylation independently of manganese status, its administration risks masking dysglycosylation and thereby depriving the clinician of glycosylation as a biomarker of adequate manganese repletion. In other words, correcting the glycosylation readout by galactose may obscure whether the underlying manganese deficiency, i.e. the true therapeutic target, has in fact been addressed, and should not be attempted where preservation of this biomarker is important for monitoring.

In conclusion, the monogenic disorders of manganese transport occupy an instructive position at the interface of metal-transport physiology and treatable neurometabolic disease, and they exemplify the reciprocal relationship between rare-disease genetics and fundamental biology. Their study has advanced the physiology of an essential yet historically underappreciated trace element, while simultaneously exposing the limits of that understanding. Continued progress will depend on the accrual of longitudinal natural-history data, the identification of phenotypic modifiers, the clarification of transporter-specific roles, and the refinement of treatment. For the field of inherited metabolic diseases, these disorders stand as a paradigm of how mechanistic insight and clinical vigilance, taken together, can convert a once-obscure metabolic pathway into an opportunity for timely diagnosis and meaningful intervention.

## Data Availability

No datasets were generated or analysed during the current study.

## Abbreviations

BBB: Blood brain barrier
CaNa_2_EDTA: Disodium calcium edetate
CDG: Congenital disorder of glycosylation
HPLC: High–performance liquid chromatography
MALDI: TOF–matrix assisted laser Desorption/Ionization Time–of–Flight
Mn: Manganese
MnSOD: Manganese superoxide–dismutase
MRI: Magnetic resonance imaging
SOD2: Superoxide dismutase 2
